# Commentary on ‘inimical effects of COVID-19 on surgical residency: Correspondence’

**DOI:** 10.1016/j.amsu.2020.09.039

**Published:** 2020-10-05

**Authors:** Hannah Jaumdally, Marwah Salih, Ahmed Al-Jabir

**Affiliations:** a King’s College London, Great Maze Pond, London, SE1 1UL, UK

## 1 Dear editor

We read with great interest the article by Yelamanchi et al. [1] regarding the challenges of exposure to surgical experience in the midst of the COVID-19 pandemic. The authors highlight several limitations to residency training including: facilities and equipment, whereby some countries may not have access to adequate animal models, simulations or cadavers to practice first hand; impact on research with reduced sample sizes and less people consenting to research; changes to learning with teaching reduced to case reports and seminars, reduced learning opportunities for junior residents in both the operating theatre and the subsequent management of surgical patients; changes to hospital processes including cancellation of elective procedures, less people attending an operating theatre at one time to reduce risk of transmission, the redeployment of surgical trainees onto COVID-related wards and the difficulties surrounding the use of personal protective equipment (PPE). Whilst this could have detrimental effects to the learning of residents and the uptake of surgical posts in the future, it has also had an impact on those even more junior: such as medical students.

The Medical Schools Council (MSC), who regulate curricula in the UK, have advised that general placements for medical students should be stopped in light of the risk to patients and to students themselves. This is due to the risk of both symptomatic and asymptomatic transmission [2]. Whilst this is enforced to protect students, it also means that many students have missed out on placements that cannot be replaced. This is particularly important in the case of paediatric and obstetrics placements – both of which have been identified by the MSC as often difficult to arrange and with limited exposure to these patient groups in other clinical specialties. Interaction with these patients is vital for the development of well-rounded medical individuals, and it is these placements that can often be the only experience a medical professional will ever have of, for example, obstetrics [3].

Furthermore, this has also impacted the education of the most junior medical trainees. Junior doctors in the UK have had their rotations suspended in April, meaning that they have missed out on four months of experience in a speciality [4].

As many trainees only have one rotation in a surgical speciality within the two-year rotational Foundation Programme, this has led to a significant number of trainees with no surgical experience beyond medical school. Additionally, this can impact both trainees with an inclination to expand their portfolio surgically and solidify career choices, as well as those interested in other specialities, where they may have to treat and diagnose surgical problems in later careers.

Overall, we wholeheartedly agree with the authors on the potential negative consequences that the COVID-19 pandemic may have on the surgical speciality and it's trainees and by extension, the repercussions on medical students, as we have identified. During this time, students are learning to adapt and manage both time and stress and should continue to maximise any knowledge they may gain from this unique and rare situation.

Provenance and peer reviewCommentary, internally reviewed.

## Author contribution

Please specify the contribution of each author to the paper, e.g. study design, data collections, data analysis, writing. Others, who have contributed in other ways should be listed as contributors.

Hannah Jaumdally – conceptualisation and manuscript writing.

Marwah Salih – conceptualisation and manuscript writing.

Ahmed Al-Jabir – conceptualisation and manuscript writing.

Guarantor.

The Guarantor is the one or more people who accept full responsibility for the work and/or the conduct of the study, had access to the data, and controlled the decision to publish. Please note that providing a guarantor is compulsory.

Hannah Jaumdally.

Marwah Salih.

Ahmed Al-Jabir.

All information contained within this manuscript is available to share and no data has been utilised.

## 2 International journal of surgery author disclosure form

The following additional information is required for submission. Please note that failure to respond to these questions/statements will mean your submission will be returned. If you have nothing to declare in any of these categories, then this should be stated.

## Please state any conflicts of interest

No conflicts of interest.

## Please state any sources of funding for your research

No funding.

## Please state whether ethical approval was given, by whom and the relevant Judgement's reference number

No ethical approval required.

## Research registration unique identifying number (UIN)

Please enter the name of the registry, the hyperlink to the registration and the unique identifying number of the study. You can register your research at http://www.researchregistry.com to obtain your UIN if you have not already registered your study. This is mandatory for human studies only.

1. Name of the registry:
2. Unique Identifying number or registration ID:
3. Hyperlink to your specific registration (must be publicly accessible and will be checked):
